# Optimal planning of integrated nuclear-hybrid renewable energy systems for electrical distribution networks based on artificial intelligence

**DOI:** 10.1038/s41598-025-11049-z

**Published:** 2025-07-17

**Authors:** Samira M. Nassar, A. A. Saleh, Ayman A. Eisa, E. M. Abdallah, Ibrahim A. Nassar

**Affiliations:** 1 Department of Nuclear Safety and Radiological Emergencies, NCRRT, Egyptian Atomic Energy Authority, Cairo, Egypt; 2 Department of Electrical Engineering, Faculty of Engineering, Al-AzharUniversity, Cairo, Egypt

**Keywords:** Nuclear energy source, Renewable energy sources, Optimization technique, Engineering, Mathematics and computing

## Abstract

In recent years, small-scale nuclear power plants, particularly micro nuclear reactors, have emerged as viable alternatives, gaining importance in the technical and economic operation of electrical distribution systems. As consumer demand for electricity continues to rise, the use of renewable energy sources and nuclear energy has become essential, especially as dependence on conventional energy sources grows increasingly unsustainable from an environmental standpoint. In this study, mathematical models for various Hybrid Energy Systems (HES) are developed using both single and multi-objective functions. Active Power Loss (APL) is selected as the first single-objective fitness function, while the total Net Present Cost (NPC) serves as the second. These two objectives are also considered together in a multi-objective optimization framework. The White Shark Optimizer is employed to determine the optimal configuration that achieves an improved voltage profile, reduces power losses, and minimizes both cost and greenhouse gas (GHG) emissions. The proposed modeling and simulations are conducted using MATLAB software, and the optimization methodology is applied to three types of HES on two standard radial distribution networks; the IEEE 33-bus and IEEE 69-bus systems. The three HES configurations analyzed are; Nuclear-Renewable Hybrid Energy System (N-R HES), Stand-alone Fossil Fuel-based Thermal Generators (FFTGs), and Renewable-Fossil Fuel Hybrid Energy System. Among the three, the N-R HES demonstrates the most favorable between system performance, cost efficiency, and environmental impact. Results and analysis prove that N-R HES is the most effective solution for sustainable energy generation and decarbonization, offering the lowest NPC and APL.

## Introduction

Electricity is essential for both economic growth and global development. As populations increase and economies advance, the demand for electricity continues to rise. In recent years, the world has encountered two significant challenges in addressing this high demand: finding ways to meet it without exhausting finite energy resources, primarily fossil fuels, and generating electricity in an environmentally responsible manner^1^.

Most electricity today is generated from traditional sources such as coal, gas, and oil. Using these resources to produce power often leads to increased greenhouse gas emissions in the environment. As a result, researchers are conducting studies to reduce the environmental impact of generating electricity from conventional sources^2,3^.

In response to growing energy demands, the world is actively seeking alternative sources of energy that can meet current needs while ensuring their availability for future generations. Renewable energy sources (RESs), like wind, ocean energy, solar, hydropower, and geothermal, are naturally abundant and are increasingly recognized as sustainable methods for generating electricity^4,5^. The weather directly impacts the availability of renewable energy sources. As a result, RES often struggle to provide a consistent energy supply over extended periods. To meet base load demands and to support RES during times of unavailability, additional energy sources are necessary. Since RESs are intermittent and long-term energy storage is not economically viable, this dependence leads to reduced energy production from RESs, thereby increasing the reliance on diesel engines to fulfill most energy demands.

As a result, the diesel engine will supply most of the energy, while renewable energy will account for only a small fraction. Consequently, integrating fossil fuel with renewable energy-dependent energy systems is not an effective strategy for reducing greenhouse gas emissions. To decrease these emissions, nuclear power is essential for electricity generation, as renewable energy sources are limited. To address the unpredictability of renewable energy, it is important to combine nuclear power with these renewable sources^6^.

Nuclear energy produces no pollutants during operation; however, some pollutants are generated during the mining, transportation, construction, and decommissioning phases. A modern alternative to fossil fuel thermal generators (FFTG) is the integration of Nuclear Power Plants (NPPs) with renewable energy sources (RES). This combination enhances the resilience, continuity, and reliability of the energy system^7^. Traditional NPPs require large installation sites and involve high initial costs. In contrast, Micro Modular Reactors (MMRs) provide a more favorable alternative by reducing capital expenses and eliminating the need for extensive installation space. MMRs offer several advantages over traditional NPPs, including shorter construction times, flexible and straightforward designs, and suitability for small-scale power production systems^8^.

The International Atomic Energy Agency (IAEA) classifies NPPs based on their power ratings. NPPs rated below 300 megawatts electric are considered “small” NPPs, while those with power ratings of up to 700 MWe are classified as "medium." Together, small and medium NPPs are often referred to as "small modular reactors" (SMRs)^9^.

The MMR (Micro Modular Reactor) is classified as a small-modular reactor, with a rating power ranging from 1 to 50 MWe. It provides a cost-effective, safe, and emission-free energy source suitable for off-grid and on-grid applications. Its compact footprint and modular design significantly influence energy system modeling. Developed in factories, the MMR enhances power generation capabilities and includes high-level safety features.

Moreover, the reactor simplifies construction, offers flexibility, and is easily transportable, requiring only a small installation area. As a result, the MMR is an ideal option for remote industries, transportation electric power, and backup power for large-scale production plants^10^.

Therefore, the necessary development of power plants will decrease by substituting FFTG with these types of micro reactors, which are currently being used with renewable energy sources either as the primary electricity-generating source or as an alternate source of power^11^.

Hence, combining renewable and nuclear energy into a single hybrid energy system can significantly enhance overall performance. This approach allows a nuclear plant to operate at full capacity while simultaneously meeting the demand for flexible generation rates. Additionally, it produces low-carbon products and energy services. By integrating renewable and nuclear energy sources, both of which emit minimal carbon dioxide during power generation, this system can effectively reduce overall carbon dioxide emissions^12^. Currently, a scientific report on nuclear-renewable integration has recently been published by the International Atomic Energy Agency (IAEA). This document explores the role of small-modular reactors in the hybridization of nuclear and renewable energy. It also addresses national goals for renewable and nuclear energy, as well as the opportunities and challenges associated with integrating these two energy sources^13^. Building on this context, the present study aims to investigate Hybrid Energy Systems (HES) through a comprehensive assessment that includes mathematical modeling, system configuration, component sizing, and performance analysis. Three different HES configurations are modeled; (1) the Stand-alone Fossil Fuel-based Energy System, (2) the Fossil Fuel–Renewable Hybrid Energy System, and (3) the Nuclear–Renewable Hybrid Energy System (N-R HES). For the first time, the White Shark Optimizer (WSO)a recent meta-heuristic algorithmis employed to optimize these configurations for solving both single and multi-objective functions.

The single-objective optimization focuses on minimizing the Net Present Cost (NPC) while also enhancing bus voltage profiles and reducing Active Power Loss (APL). Meanwhile, the multi-objective optimization simultaneously targets the minimization of NPC and APL, along with improvements in voltage profile. This multi objective optimization strategy represents a novel contribution, as it addresses both technical and economic aspects, unlike many prior studies that primarily emphasize economic performance indicators.

A comparative performance analysis of the three HES configurations is performed using multiple techno-economic key performance indicators (KPIs), including APL, greenhouse gas (GHG) emissions, and NPC. The simulation is performed on two standard radial distribution test systems: the IEEE 69-bus and IEEE 33-bus networks. Results indicate that the Nuclear–Renewable Hybrid Energy System (N-R HES) outperforms the other configurations, offering significant reductions in GHG emissions while enhancing both technical and economic viability.

## System modeling

This study evaluates the costs associated with Diesel Generators, Micro Modular Reactors (MMRs), and Renewable Energy Sources (RES), while also accounting for active power losses to improve overall network performance. It considers both the financial aspects of these energy systems and the practical constraints involved in their implementation. The detailed steps of the study are outlined in the supplementary material titled "Overview of Study Steps".

The analysis focuses on three system configurations:

- Fossil fuel and renewable hybrid energy systems
- Standalone fossil fuel energy systems
- Nuclear–renewable hybrid energy systems (N-R HES)

Key performance indicators (KPIs) related to cost and power losses for each configuration are modeled using MATLAB 2020. The White Shark Optimizer (WSO) algorithm is utilized to optimize all systems, with the primary objective of identifying the configuration that minimizes both Active Power Loss (APL) and Net Present Cost (NPC).

### Solar energy

The solar power output from solar PV is influenced by ambient temperature, the surface area of the solar PV system, and solar irradiance ($\text{SR})$^14,15^.The solar PV power generation is determined using the following equations:

1 $${\text{p}}_{\text{PV}}\left(\text{t}\right)={\text{NPV}\times \text{p}}_{\text{R},\text{PV}}\times \left(\frac{\text{SR}}{{\text{SR}}_{\text{ref}}}\right)\times \left[1+{\text{N}}_{\text{T}}\left({\text{T}}_{\text{C}}-{\text{T}}_{\text{ref}}\right)\right]$$

2 $${\text{T}}_{\text{C}} ={\text{T}}_{\text{air}}+\left(\left(\frac{{\text{T}}_{\text{NO}}-20}{800}\right)\times \text{SR}\right)$$

where,${\text{p}}_{\text{R},\text{PV}}$, ${\text{SR}}_{\text{ref}}$, ${\text{N}}_{\text{T}}$, and NPV denote the rated power of the PV panel, reference solar radiation (1000 W/m^2^), module temperature coefficient (− 3.7 × 10^3 (1/°C)), and the number of PV panels, and ${\text{T}}_{\text{ref}}$ indicates the reference (25°C),${\text{T}}_{\text{NO}}$ and ${\text{T}}_{\text{air}}$ are normal and ambient operating cell temperature, respectively. Technical specifications of the solar PV module utilized in the study are provided in Table 1^10^.

**Table 1:** The technical specifications of solar photovoltaic (PV) systems.

Characteristics	Values
Capital cost ($/kW)	640
Lifetime (years)	25
Efficiency of the MPPT unit (%)	100
O&M cost ($/kW)	640
Reference efficiency of PV panel (%)	24
Nominal operating cell temperature (°C)	45
Replacement cost ($/kW/Year)	12
PV panel reference temperature(°C)	25
Temperature coefficient (1/°C)	0.0041

### Wind power

One effective energy source that can generate electricity without using fuel is a wind turbine generator. The output power of a wind turbine can be calculated using the following formula^16^:

3 $${\text{P}}_{\text{W}}\left(\text{t}\right)=\left\{\begin{array}{c}0 V<{\text{V}}_{\text{cin}} ,V>{\text{V}}_{\text{out}}\\ {\text{P}}_{\text{r}}\times \left(\frac{\text{V}\left(\text{t}\right)-{\text{V}}_{\text{cin}}}{{\text{V}}_{\text{r}}-{\text{V}}_{\text{cin}}}\right){\text{V}}_{\text{cin}}\le V\left(\text{t}\right)\ge {\text{V}}_{\text{r}} \\ {\text{ P}}_{\text{r}}{\text{ V}}_{\text{r}}\le V\left(\text{t}\right)\ge {\text{V}}_{\text{out}}\end{array}\right.$$

where,${\text{P}}_{\text{r}}$, and ${\text{P}}_{\text{W}}\left(\text{t}\right)$ denote the wind turbine’s rated power (kW) and the power generated (kW) at each time step (t), respectively., ${\text{V}}_{\text{r}}$ indicates the calculated wind speed (m/s) at the hub height at “t” step time and, ${\text{V}}_{\text{cin}}$ is cut-in speed of the wind turbine, $\text{V}\left(\text{t}\right)$ is the wind turbine rated speed (m/s)and ${\text{V}}_{\text{out}}$ is cut-out speed (m/s), sequentially.The technical specifications of the wind turbine used in the study are presented in Table 2^16^.

**Table 2:** The technical Specifications of wind turbine generator.

Characteristics	Values
Nominal capacity (kw)	3600
Lifetime (years)	25
Capital cost ($/kW)	1130
Cut-in speed (m/s)	3.5
Anemometer height (m)	50
Rated speed (m/s)	12
Hub height (m)	45
Cut-out speed (m/s)	25
O&M cost ($/kW/Year)	48
Power law exponent	1/7
Replacement cost ($/kW)	1130

### Diesel generator

This study compares the proposed N-R HES with traditional energy systems that rely on fossil fuel-based generators (FFG). The costs associated with a diesel generator can be generally classified into three main parameters: capital cost, maintenance cost, and operating cost. The decommissioning cost is considered negligible^17^. The technical specifications of the diesel generator are presented in Table 3^18^.

**Table 3:** The technical specifications of the diesel generator.

Characteristics	Values
Generator size (kw)	1000
Lifetime (years)	2.5
Capital cost ($/kW)	800
Fuel cost ($/kWh)	202
O&M cost ($/kW/Year)	35
CO_2_ emissions (kg /MWh)	700

### Small and micro modular reactors

SMRs are nuclear reactors of the fourth generation, capable of producing up to 300 megawatts of power. Within this category, MMRs are a type of small-scale fourth-generation nuclear reactor with outputs ranging from 1 to 50 megawatts electrical. MMRs can utilize Combined Heat and Power (CHP) systems, allowing them to generate both thermal energy and electricity simultaneously. They have the potential to serve as a reliable source of electricity in isolated locations not connected to any electrical grid. Several companies are actively developing small and micro-scale nuclear reactors, and there are several advantages of MMRs over conventional large-scale nuclear power plants. First, MMR designs prioritize safety and ease of use, incorporating built-in safety features, sealed cores, and modular construction. They are designed to be simple to operate and can be constructed quickly. MMRs are produced at a factory, then packaged and transported to their designated locations. Some designs are self-contained and require minimal human intervention. The scale and risk associated with MMRs are similar to those of research reactors, which have a long history of safe operation^19^.

The initial deployment of a new technology typically incurs higher installation costs compared to subsequent deployments. As experience is gained and lessons are learned, these costs tend to decrease. The experience gained from operating production plants, is referred to as the learning rate. The "one-factor learning curve" formula can be used to illustrate the relationship between the lessons learned and the reduction in technology costs^18^:

4 $$LR=1-{2}^{R}$$

where, $\text{R}$ denotes the cost reduction rate (%) and $\text{LR}$ presents the learning rate. The actual rate of learning varies from case to case. The location and complexity of a project’s design determine the unique costs associated with it, including the fixed costs of equipment related to a specific learning rate. As the learning rate increases, the overnight capital cost of the MMR units decreases. The capital costs associated with multiple units can be determined using the following formula^18^:

5 $${CT}_{u}={CT}_{1st}\times {N}_{u}^{R}$$

where, $${\text{CT}}_{\text{u}}$$ is the MMR unit cost of $$\text{Nu}$$ number unit ($), and $${\text{CT}}_{1\text{st}}$$ is the 1st MMR unit cost ($). Table 4 shows the detailed MMR input parameters^19^.

**Table 4:** Technical specifications of MMR.

Characteristics	Values
Reactorsize (kWe)	1000
Lifetime (years)	40
Capital cost ($/kWe)	15,000
lifetime of the core (years)	10
fuel Cost ($/MWh)	10
CO_2_ emissions (kg /MWh)	4.55
O&M Cost ($/kWe)	350
Capacity factor (%)	95
Refueling cost of fuel module (million $)	20
Plant efficiency (%)	40
Decommissioning cost ($/MWh)	5

As the MMRs are produced in the factory, it is expected that the learning curve will be between 5 and 15%. For this study, an average learning rate of 10% is considered. Additionally, it is anticipated that as more operational experience is gained, both fuel costs and operations and maintenance (O&M) costs will decrease. However, the analysis excludes the reduction in O&M and fuel costs to avoid unnecessary complications, as these expenses constitute only a small portion of the overall costs. The primary factor influencing the value of MMR is the overnight expenses.

In the MMR capital cost study, both licensing costs and site engineering costs are included. Due to the variety of manufacturers and technologies, refurbishment costs may not be factored into a fixed economic model for MMRs. Therefore, refurbishment costs are instead included in the fixed O&M cost.

Decommissioning costs accrue while the MMR is in operation, and these costs are regarded as being equally distributed over the project’s duration.

The total cost associated with transporting the fuel module from the factory to the designated site, as well as installing it, is known as the refueling cost. The MMR fuel cost is deducted from the refueling cost since it is already included in the overall fuel costs.

Nuclear power plants, including microreactors, can operate in two different modes: base load and load-following. In base load mode, the microreactor (MMR) consistently delivers its maximum power level. In contrast, a load-following microreactor adjusts its output based on short-term or long-term variations in system demand.

When traditional base load systems, like NPPs, are adapted to manage fluctuating demand, it increases wear and tear on the system and raises O&M costs. The amount of electricity generated does not affect the costs of fuel or O&M. Consequently, load-following, which can lead to reduced electricity output, is considered uneconomic and very inefficient^19^.

In contrast, base-load mode operates simply and efficiently, consistently supplying a specific quantity of energy over a given period. Variable renewable energy sources and dispatchable generating sources provide for the remaining demand in HES. The ideal configuration, availability, and overall system cost all influence a suitable energy mix. Furthermore, load-following NPPs are necessary if nuclear generation accounts for a significant portion of energy contribution. The overall contribution of nuclear energy to HES is diminished when combined with renewable energy sources. This research focuses on the base-load operation of MMRs, examining all of the aspects mentioned above ^18^.

## Key performance indicators (KPIs)

The feasibility is assessed by comparing various energy systems using the KPIs. The following economic, technical, and environmental KPIs are used in this study.

### Economical KPIs (net present cost)

The primary distinction between Net Present Value (NPV) and Net Present Cost lies in their respective signs. NPV represents all future cash flows present value associated with an investment, including both positive and negative amounts, calculated using a discount rate. For investors, a lower NPC indicates a greater potential profit^20^. The NPC can be determined using the following formula.

6 $$\text{Net Present Cost }\left(\text{NPC}\right)=-\text{ Net Present Value }(\text{NPV})$$

7 $$\text{NPV }=\frac{\text{Cashflow}}{{(1 +\text{rld})}^{\text{t}}}-\text{ Initial Investment}$$

8 $$rld=\frac{i-f}{1+f}$$

where, rld denotes the real discount rate (%), f is nominal discount rate (8%), i indicates inflation rate (2%), and t is the number of the time periods, respectively. This analysis takes into account the real discount rate by considering the impact of inflation^21^. The following formula can be used to determine the NPC over lifetime project.

9 $$NPV=\sum_{t=0}^{n}\frac{{R}_{t}}{{(1+rld)}^{t}}$$

where, n denotes the project lifetime and ${R}_{t}$ indicate the inflow and outflow of net cash over a specific time period.

### Technical KPIs (active power loss)

Loss minimization is one of the key operational prerequisite in RDS for improving the efficient use of (WTG and PV) energy^22^. In this study backward-forward sweep approach is performed for load flow solution^23^.

10 $${\text{P}}_{\text{Tloss}}=\sum_{{\text{K}}_{(\text{mn})}=1}^{{\text{n}}_{\text{br}}}{\text{R}}_{(\text{K})}\left[\frac{{\text{P}}_{(\text{n})}^{2}+{\text{Q}}_{(\text{n})}^{2}}{{\left|{\text{V}}_{(\text{n})}\right|}^{2}}\right]$$

where, n_br_ denotes the branches number of network, k is the index branch between buses m and n,${\text{P}}_{\text{Tloss}}$ is the active power loss, P(n) is the load real power, Q(n) is load reactive power, V(n) is the magnitude of the voltage at nth bus.

### Environmental KPIs (CO_2_ gas emissions)

Energy producers release various pollutants over their operational lifetime, including sulfur dioxide nitrogen oxides, particulate matter,carbon monoxide, unburned hydrocarbons (UHC), and carbon dioxide. This study focuses specifically on carbon dioxide (CO_2_) emissions. The following formula calculates the amount of CO_2_ produced by any generator^12^.

11 $$\text{CO}2\text{ emissions }=\text{ Emission Factor }\left(\frac{kg}{MWh}\right)\times \text{ AGE }\left(\text{MWh}\right)$$

where, AGE is the annually generation of electricity of the generators. The SMRs emissions factor and diesel generators are 4.55 (kg/MWh) and 700 (kg/MWh), respectively^24^. When calculating the NPC, the CO_2_ emissions penalty is taken into consideration. The following formula can be utilized to determine the annual penalty for CO_2_ emissions.

12 $$CCE=ACE\times CEP\times \frac{i{(1+rld)}^{n}}{{(1+rld)}^{n}-1}$$

where, $$\text{CCE}$$ is The cost of the penalty for CO_2_ emissions ($),$$\text{CEP}$$ and $$\text{ACE}$$ arethe penalty of CO_2_ emissions ($/tonne) and the CO_2_ emissions annually (tonne). Carbon taxes differ significantly across countries. The International Monetary Fund (IMF) has determined that for major CO_2_emitting nations to fulfill their carbon emission reduction commitments, they should implement a charge of between $50 and $100 per ton by 2030. For this analysis, a CO_2_ emissions penalty of $30 per ton is used to demonstrate its impact on the NPC.

## Problem formulation

The optimization problem formulation, involving the objective functions and constraints is discussed in this section using the WSO algorithm.

### Objective function

The optimization problem seeks to identify the best configuration for HES to achieve minimizing in both APL and NPC. In addition to the N-R HES, two other energy systems are optimized for comparison: the Renewable and Fossil Fuel Hybrid Energy System and the Fossil Fuel-based Energy System.

The objective function of the optimization problem discusses the economical and technical KPIs, as well as its constraints represent environmental KPI. The total APL for test system (IEEE 33 bus) when integrated different Energy System acts as the first fitness function, while the total NPC of each energy system acts as the second fitness function. This approach clearly outlines the fitness functions used to express the optimization problem as follow:

13 $$\text{min}{f}_{APL}=\sum {P}_{Tloss}$$

14 $$\text{min}{f}_{NPC}=\sum_{j\epsilon k}{NPC}_{j}$$

where, ${NPC}_{j}$ is the NPC of the ${j}^{th}$ component, while $k$ refers to the set of energy system components. The optimal balancing solution for MOF is determined using the weighted sum method^25^, as shown in Eq. (15):

15 $$\text{minf}={\text{w}}_{1}\frac{{\text{P}}_{\text{L}}^{\text{with DG}}}{{\text{P}}_{\text{L}}^{\text{no DG}}}+{\text{w}}_{2}\frac{{\text{NPC}}^{\text{with DG}}}{{\text{NPC}}^{\text{no DG}}} , {\text{w}}_{1}+{\text{w}}_{2}=1$$

where, ${\text{w}}_{1}$ and ${\text{w}}_{2}$ are the weighting factors of active power loss and NPC, respectively; ${\text{P}}_{\text{L}}^{\text{with DG}}\text{ and}{\text{ NPC}}^{\text{with DG}}$ indicate APL and NPC after energy sources installation; ${\text{P}}_{\text{L}}^{\text{no DG}}\text{ and }{\text{NPC}}^{\text{no DG}}$ denote APL and NPC before energy systems integration.

For the two objectives, the fuzzy member ship functions are computed as follows:

16 $${\text{f}}_{\text{PL}}\left(\text{p}.\text{u}\right)=\frac{{\text{P}}_{\text{Lmax}}-{\text{P}}_{\text{L}}}{{\text{P}}_{\text{Lmax}}-{\text{P}}_{\text{Lmin}}}$$

17 $${\text{f}}_{\text{NPC}}\left(\text{p}.\text{u}\right)=\frac{{\text{NPC}}_{\text{max}}-\text{NPC}}{{\text{NPC}}_{\text{max}}-{\text{NPC}}_{\text{min}}}$$

where,${\text{P}}_{\text{Lmin}},{\text{P}}_{\text{Lmax}}$ indicatethe minimum and the maximum value of total power loss, respectively;${\text{P}}_{\text{L}}$ indicates to the APL value;${\text{NPC}}_{\text{max}},{\text{NPC}}_{\text{min}}$ denote the maximum and the minimum value of NPC, respectively.

The energy system consists of a diesel generator, MMR, WTG, and solar PV. The net present value of each component is the current value of all associated costs, which include capital cost, operating and maintenance costs, replacement costs, and fuel expenses. Additionally, the costs of decommissioning the MMRs and refueling cost are also factored. A formula for calculating the NPC of any energy system component is discussed below.

18 $${NPC}_{j}={C}_{cap,j}+{C}_{O\&M,j}+{{C}_{fuelc,j}+C}_{rep,j}-{C}_{salv,j}$$

where, ${\text{C}}_{\text{cap},\text{j}}$, ${\text{C}}_{\text{O}\&M,j}$, ${\text{C}}_{\text{fuelc},\text{j}}$,${\text{C}}_{\text{rep},\text{j}}$ and ${\text{C}}_{\text{salv},\text{j}}$ refer to the current value of capital cost, O&M cost, fuel cost, replacement cost, and the salvage value of the ${j}^{th}$ component, respectively. The salvage value represents the amount of value that remains at the end of the project lifecycle when the component is no longer in use. At the start of the project, the capital cost for each component is determined. Both the capital cost of each component and the total number of components are factored into the overall capital cost. The following formula can be used to calculate the capital cost:

19 $${C}_{cap,j}={N}_{com,j}{\times C}_{capc,unit(j)}$$

where, ${C}_{cap,j}$ represents the capital cost of the ${j}^{th}$ component, ${N}_{com,j}$ is the components number, and ${C}_{capc,unit(j)}$ refers to the cost of the ${j}^{th}$ unit. MMR capital costs are calculated differently because the cost reduction is included as part of the overall capital cost. Furthermore, the rate at which MMRs reduce their costs is correlates with their learning rate. The following formula can be utilized to calculate the capital cost of MMRs^12^:

20 $${C}_{cap,MR}=\sum_{k=1}^{{N}_{MR}}{C}_{capc,MR(1st)}\times {\left({N}_{MR}\right)}^{R}$$

where,${C}_{cap,MR}$ refers to the total MMR capital cost,${C}_{capc,MR(1st)}$ implies theprice ofthe ${1}^{st}$ MMR unit, ${\text{N}}_{\text{MR}}$ is theMMR numbers,and $\text{R}$ represents thecost reduction rate. Operating and Maintenance costs for a component are incurred annually and continue until the project’s completion. Each year, the O&M costs of components are determined using the following formula^12^.

21 $${C}_{O\&M,j}={{N}_{com,j}\times C}_{O\&M,yearly(j)}\times \frac{{(1+rld)}^{n}-1}{rld{(1+rld)}^{n}}$$

where,${C}_{O\&M,j}$ denotes the present value of the overall O&M cost, ${N}_{com,j}$ is the components number, and ${C}_{O\&M,yearly(j)}$ indicates the annual O&M cost of the ${j}^{th}$ component. Any component that has reached the end of its lifespan needs to be replaced. The number of replacements required is determined by the project overall lifespan and the lifetime of the individual components. To calculate the present value of the replacement costs for these components, the following formula is used^12^:

22 $$NR=ceil\lceil\frac{n}{{CLT}_{j}}\rceil-1$$

23 $${F}_{rep}=\sum_{k=1}^{NR}(k{\times CLT}_{j})$$

24 $${C}_{rep,j}={N}_{com,j}\times {C}_{rep,unit(j)}\times \frac{1}{{(1+rld)}^{{F}_{rep}}}$$

where,${C}_{rep,j},{CLT}_{j}$, NR, and ${C}_{rep,unit(j)}$ refer to the present value of the ${j}^{th}$ component replacement cost, the ${j}^{th}$ unit lifetime, NR is the required number of replacement, and the per-unit replacement cost of the ${j}^{th}$ component, respectively.The function ceil(X) rounds the value of X up to the nearest whole number that is equal to or greater than X. When calculating costs for MMRs (Multi-Modal Resources) and fossil fuel generators, the price of fuel is factored in. In contrast, renewable energy sources like wind turbines and solar photovoltaic do not require fuel. The annual fuel cost is calculated using a specific formula^12^:

25 $${C}_{fuelc,j}={E}_{yearly,j}\times {CU}_{fuel,j}\times \frac{{(1+rld)}^{n}-1}{rld{(1+rld)}^{n}}$$

where,$${C}_{fuelc,j}$$ denotes the $${j}^{th}$$ component fuel cost ($),$${E}_{yearly,j}$$ represents the $${j}^{th}$$ component energy generation annually (MWh),$${CU}_{fuel,j}$$ the perunit fuel price energy generation of the $${j}^{th}$$ component, and $${RAT}_{j}$$ isthe $${j}^{th}$$ component rating. The salvage value is calculated based on linear depreciation. When a component’s salvage value is directly proportional to its remaining lifespan, this is referred to as linear depreciation. The following formula is used to determine the present value of the salvage value^12^:

26 $${C}_{salv,j}={N}_{com,j}\times {C}_{rep,unit(j)}\times \frac{{CLT}_{rem,j}}{{CLT}_{j}}\times \frac{1}{{(1+rld)}^{n}}$$

27 $${CLT}_{rem,j}={CLT}_{j}-\left(n-{LT}_{rep,j}\right)$$

28 $$LT_{rep,j} = CLT_{j} \times floor\left\lfloor {\frac{n}{{CLT_{j} }}} \right\rfloor$$

where,${C}_{salv,j}$ represents the present worth of the ${j}^{th}$ component of the salvage value, and ${CLT}_{rem,j}$ indicates the ${j}^{th}$ component remaining life at the end of the project lifetime. The function floor(X) rounds the number X down to the nearest whole number that is less than or equal to X. This analysis focuses solely on MMRs when calculating the costs associated with decommissioning and refueling. It includes a yearly distribution of decommissioning costs, even though the actual decommissioning of MMRs takes place at the end of the project^12^.

29 $${C}_{decom,MR}={E}_{yearly,MR}\times {CU}_{decom,MR}\times \frac{{(1+rld)}^{n}-1}{rld{(1+rld)}^{n}}$$

where,$${C}_{decom,MR}$$ denotes the MMR total decommissioning cost($),$${\text{E}}_{\text{yearly}}$$, _MR_ represents the annually MMR energy generation (MWh), and $${C}_{decom,MR}$$ refers totheper unit decommission cost($/MWh). In this study, the fuel module lifetime is established at ten years, indicating that the MMR will be refueled every decade. Fuel costs are not included in the overall refueling expenses for the MMRs. The refueling costs consist of labor costs, gasoline transportation costs, and other related expenses. The present value of the refueling costs is calculated using the following formulas^12^.

30 $${C}_{refueling,MR}={N}_{MMR}\times \sum_{k=1}^{{MR}_{refuel}}{C}_{refueling,MR(unit)}\times \frac{1}{{(1+rld)}^{{F}_{refuel}}}$$

31 $${MR}_{refuel}=ceil\lceil\frac{n}{{LT}_{fb(MR)}}\rceil-1$$

32 $${F}_{refuel}=\sum_{k=1}^{M{R}_{refuel}}(n{\times LT}_{fb(MR)}$$

where,${C}_{refueling,MR}$ indicates the MMR refueling cost,${C}_{refueling,MR(unit)}$ refers to the cost of refueling every decade,${LT}_{fb(MR)}$ denotes the fuel bundle lifetime, and ${MR}_{refuel}$ represents the required refueling number in the lifetime project.

### Constraints

In order to solve the proposed objective functions, the following constraints must be met^26^.

Power balance constraint:

33 $$\sum_{\text{n}=1}^{{\text{N}}_{\text{bus}}}{\text{P}}_{\text{DGn}}=\sum_{\text{n}=1}^{{\text{N}}_{\text{bus}}}{\text{P}}_{\text{D},\text{n}}+{\text{P}}_{\text{Tloss}},\sum_{\text{n}=1}^{{\text{N}}_{\text{bus}}}{\text{Q}}_{\text{DGn}}=\sum_{\text{n}=1}^{{\text{N}}_{\text{bus}}}{\text{Q}}_{\text{D},\text{n}}+{\text{Q}}_{\text{Tloss}}$$

Voltage magnitude constraint:

34 $${\text{V}}_{\text{n}}^{\text{min}}\le {\text{V}}_{\text{n}}\le {\text{V}}_{\text{n}}^{\text{max}}$$

DG size constraint:

35 $${\text{P}}_{\text{DGn}}^{\text{min}}\le {\text{P}}_{\text{DGn}}\le {\text{P}}_{\text{DGn}}^{\text{max}} , {\text{Q}}_{\text{DGn}}^{\text{min}}\le {\text{Q}}_{\text{DGn}}\le {\text{Q}}_{\text{DGn}}^{\text{max}}$$

where,${N}_{bus}$ indicates the buses number, ${P}_{DGn}$ and ${Q}_{DGn}$ refer to the available active and reactive power as a result of the DG placement at bus n, ${P}_{Dn}$ and ${Q}_{Dn}$ are the active and reactive power demands at bus n, ${V}_{n}^{min}$ is the minimum voltage bound and ${V}_{n}^{max}$ is the maximum bound of system voltage^27^.

### Implementation of optimization algorithm: white shark optimizer

The literature indicates that researchers have employed both artificial intelligence (AI)-based and conventional optimization techniques. Traditional methods, while grounded in initial assumptions that make them user-friendly and capable of producing meaningful results, often face limitations due to the restrictive nature of those assumptions. These limitations must be carefully addressed to ensure accuracy and reliability. In the context of distribution systems, effective allocation of energy sources is critical; hence, the application of heuristic and meta-heuristic optimization techniques is highly recommended.

The White Shark Optimizer (WSO), first introduced in 2022, offers several advantages for solving global optimization problems. It is recognized for its flexibility in handling a wide variety of optimization challenges, along with its simplicity, robustness, and ability to efficiently converge on global solutions. Notably, WSO exhibits a high convergence rate even in complex problem spaces. Another significant advantage of WSO is its ability to deliver practical and cost-effective solutions for complex optimization scenarios, with minimal need for parameter adjustments making it a versatile tool for a broad range of applications. The mathematical model used for WSO initialization, iteration, and halting phases is covered in this section^28^.

The “n” white shark population, along with its locations in the problem area, suggests a potential solution. The regular random initialization discussed below is used to create the initial population in the search domain.

37 $${w}_{j}^{i}={l}_{j}+ \times \text{r}({u}_{j}-{l}_{j})$$

where,${w}_{j}^{i}$ denotes the ith white shark initial vector in the jth dimension,${{u}_{j}andl}_{j}$ indicate the upper and lower search space boundaries in the jth dimension, respectively and r is a random number created in the interval [0, 1].

Simultaneously, the white sharks adjust their posture in a wavy motion towards the prey, as illustrated by Eq. (37).

37 $${u}_{k+1}^{i}=\mu \left[{u}_{k}^{i}+{p}_{1}\left({w}_{gbestk}-{w}_{k}^{i}\right)\times {c}_{1}+{p}_{2}({w}_{best}^{{v}_{k}^{i}}-{w}_{k}^{i})\times {c}_{2}\right]$$

where,${u}_{k+1}^{i}$ indicates the new ith white shark speed vector in the iteration (k + 1)th, i = 1, 2, . . ., n, is the index in population size “n” of white shark, ${u}_{k}^{i}$ denotes the i^th^ white shark present velocity vector in the kth step, ${w}_{k}^{i}$ is the ith white shark present position vector in the iteration (k)^th^, ${w}_{best}^{{v}_{k}^{i}}$ is the ith optimum position vector that the swarm recognizes, ${w}_{gbestk}$ refers to the optimal location vector in the kth step that any white shark has yet to create, p_1_ and p_2_ are two white shark strength c_1_ and c_2_ denote the numbers generated uniformly consistently in the interval [0, 1], and ${v}_{i}$ is the ith white shark index vector that reached the optimal position given by Eq. (38).

38 $$\nu = \left\lfloor {n \times rand(1,n)} \right\rfloor + 1$$

Now, the behavior of white sharks when approaching prey was described using the location update mechanism detailed in Eq. (39). Thus, great white sharks can sustain their position in the optimal location closest to their prey. This behavior is described in Eq. (40).

39 $${w}_{k+1}^{i}=\left\{\begin{array}{c}{w}_{k}^{i}.{w}_{o}+u.a+l.b,rand<mv\\ {w}_{k}^{i}+\frac{{u}_{k}^{i}}{f} ,rand\ge mv\end{array}\right.$$

40 $$w_{k + 1}^{\prime i } = w_{gbestk} + r_{1} \mathop{\longrightarrow}\limits_{{D_{w} }}^{}{\text{sgn}} (r_{2} - 0.5)r_{3} < s_{s}$$

where, ${w}_{k+1}^{i}$ indicates the ith white shark updated vector site at (k + 1)th step, b and a represent a single-dimension binary number, and l and u refer to the search space of the lower and upper boundaries, respectively. The variables "$mv$ " and "$f$" denote the motion energy and frequency of a white shark, respectively, “rand” denotes a random number generated in the interval from [0, 1], and w_o_ represents a logical vector.

**Fig. 1:**
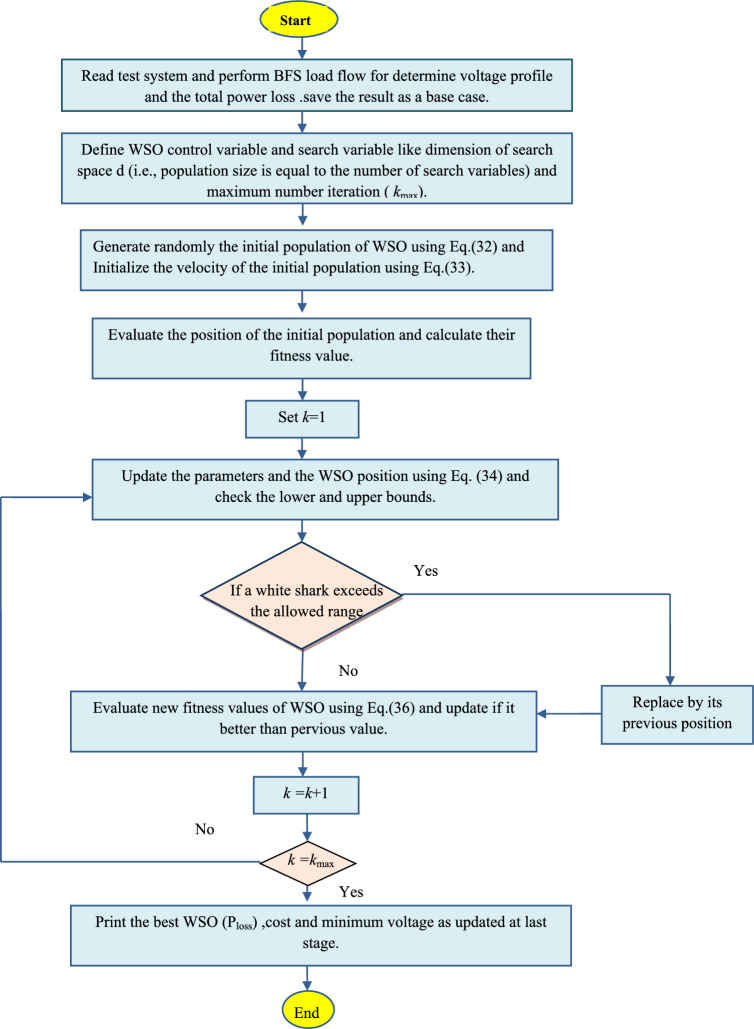
Flow chart of the implemented WSO algorithm.

The flowchart in Fig. 1 illustrates the implemented WSO method.

## Results and discussion

This section analyzes the results of the study. It compares the proposed energy systems using technical, financial, and environmental key performance indicators. Performance analysis of the IEEE 33 and IEEE 69 bus systems demonstrates the effectiveness of the proposed algorithm^29^.The first test system, the IEEE 33-bus, has a total load of 3.72 MW and 2.3 MVAR^30^. Detailed data for this system are provided in Supplementary Appendix A. The second test system, the IEEE 69-bus, has a total load of 3.89059 MW and 2.6936 MVAR at a voltage of 12.6 kV^31^. Additional details are available in Supplementary Appendix B.

### IEEE 33-bus radial distribution system

Several case studies were simulated using the WSO algorithm, focusing on power losses, net present cost, voltage profiles, and greenhouse gas emissions. Three different scenarios were designed to explore a variety of case studies, each with distinct objective functions and constraints. The following provides a description of these scenarios:

- Scenario 1: Active Power Loss Minimization
- Scenario 2: Minimization Net Present Cost
- Scenario 3: Multi-objective function (minimizing net present cost, and active power loss).

#### Scenario1: active power loss minimization (single-objective problem)

The WSO algorithm is utilized to achieve optimal allocation among the three energy systems, through solving a single objective problem which is minimize total active power loss. The three energy resources are described as follows:

- Case-01: Stand alone Fossil Fuel Energy Systems,
- Case-02: Fossil Fuel and Renewable Hybrid Energy Systems, and.
- Case-03: Nuclear Renewable Hybrid Energy Systems.

The consequences of implementing the three cases in each of the previously mentioned scenarios are analyzed by observing various parameters, including unit positions, size, voltage magnitude, and active power loss reduction using WSO. For the research on renewable energy systems, a single day representative wind speed and solar radiation of daily summer in the Upper Egypt region is provided, as illustrated in Table 5^32^.

**Table 5:** Daily summer wind speed and solar radiation.

Time (h)	Solar radiation (w/m^2^)	Wind speed (m/s)
0	0	5.5
1	0	5.1
2	0	4.6
3	0	4.0
4	0	4.2
5	0	4.3
6	14	4.8
7	63	4.4
8	172	4.3
9	395	4.1
10	653	4.3
11	849	4.5
12	979	4.8
13	1020	4.9
14	978	5.3
15	856	6.2
16	663	7.1
17	417	7.9
18	184	8.2
19	49	8.6
20	2	7.5
21	0	6.8
22	0	5.9
23	0	5.6

**Fig. 2:**
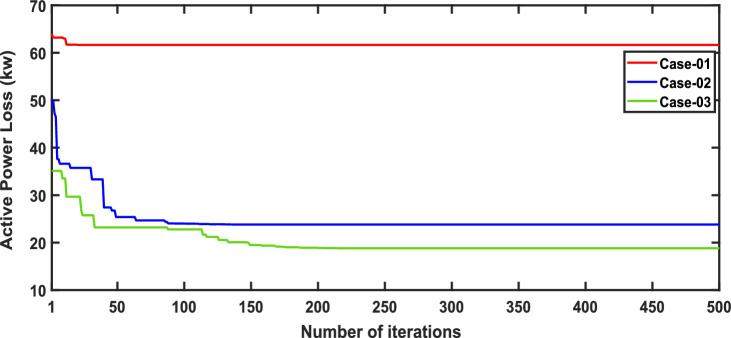
Convergence characteristics of IEEE 33-bus for scenario 1 for Case-01, Case-02 and Case-03.

**Fig. 3:**
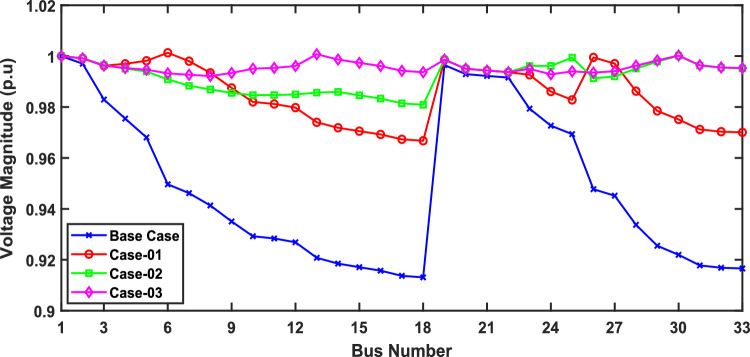
Voltage profile for first scenario of IEEE 33-bus under different case studies.

In scenario 1, the simulation was performed for three cases including case-01 (FFTGs), case-02 (FFTGs and RESs), and case-03 (N-R HES), respectively; according to Table 6 in the base case, the minimum voltage is 0.9131 p.u., and the active power loss is 202.66 kW. In the first case, by integrating FFTGs, the reduction of APL is70%, and the minimum voltage is 0.967. The cost in this case is 53 million dollars, and CO_2_ emissions are 16,082 tons/year with a penalty of $49,886. In the second case, by integrating FFTGs with RESs, APL reduces to 23.8 KW with a reduction of 88%, and the minimum voltage is 0.981. The corresponding cost in this case is 29 million dollars, and CO_2_ emissions are 8196 tons/year with a penalty of $25,423. In the third case, for the implementation of N-R HES, APL decreases to 18.81 KW with reductions of 91% and the minimum voltage to 0.992. The resulting cost is 46million dollars, and CO_2_ emissions are 49.8 tons/year with a penalty of $154.3. The comparisons between the effects of the integration of different energy systems using the suggested approach are shown in Table 6. Figure 2 presents the convergence graph comparison of all cases for this scenario. Also, a voltage profile comparison for all cases is shown in Fig. 3. It’s observed that among the three cases, Case-01 has the lowest APLR at 70%, while Case-03 has the highest APLR at 91%. This improvement in Case-03 can be attributed to the integration of the renewable and nuclear energy sources, as both the diesel SMRs and wind turbine contribute to providing active and reactive power which results in a more significant minimization in active power loss. The least environmental impact is observed in Case-03; N-RHES emits only 49.8 tons of CO2 annually, whereas Case-01 emits 16,082 tons. Consequently, the penalty for CO2 emissions is highest for Case-01 and lowest for Case-03. Therefore, Case-03 outperforms the other two energy systems in the IEEE 33-bus radial distribution network regarding both technical (APL) and environmental (GHG emissions) KPIs.

**Table 6:** Optimization results for first scenario of IEEE 33-bus for various energy systems.

Parameter	Base Case	Case-01	Case-02	Case-03
APL (KW)	202.7	61.66	23.81	18.81
APL reduction (%)	–	70	88	91
Cost (million $)	–	53.52	29.36	46.29
Generator/MMR(MW), Loc	–	2.6 (6)	1.336 (30)	1.248 (30)
Generator (MVAR)	–	1.63	0.828	0.774
Solar PV(MW), Loc	–	0.00	0.780 (25)	0.780 (25)
Wind (MW), Loc	–	0.00	0.691 (14)	0.753 (13)
Wind (MVAR)	–	0.00	0.428	0.466
V_MIN_ (p.u.)	0.9131	0.967	0.981	0.9922
CO_2_ emission(ton/year)	–	16,082	8196	49.75
CO_2_ Penalty ($)	–	49,886	25,423	154.32

#### Scenario 2: optimizing single-objective problem (net present cost (NPC))

**Fig. 4:**
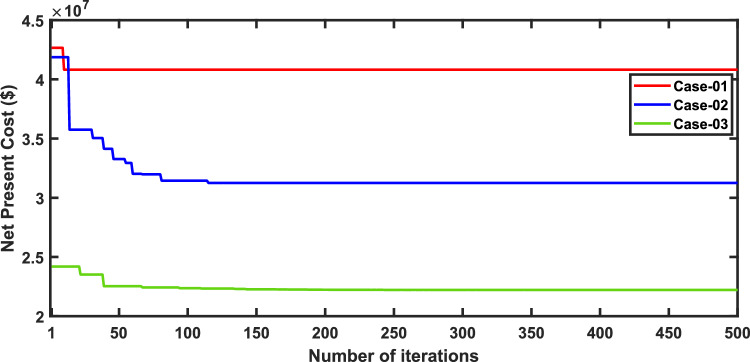
Convergence characteristics of IEEE 33-bus for scenario 2 for Case-01, Case-02 and Case-03.

**Fig. 5:**
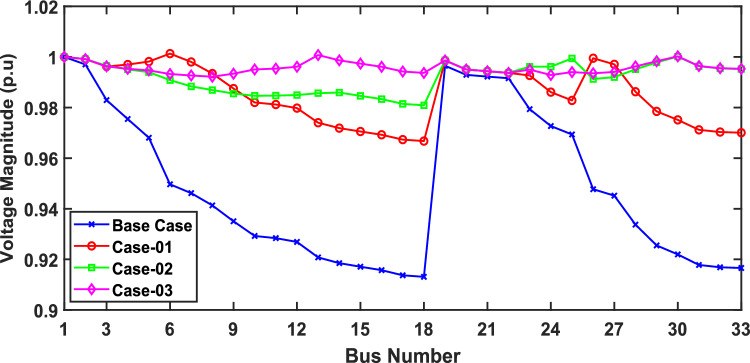
Voltage profile for second scenario of IEEE 33-bus under different case studies.

In this scenario, the net present costs of the three energy systems were optimized by using the optimal distribution of DG, PV, WTG, and MMR units. Table 7 compares each case effect using the suggested approach. Furthermore, the convergence characteristic of the recommended approach in this case is displayed in Fig. 4. The voltage profile of the energy systems integration is displayed in Fig. 5.

**Table 7:** Simulation results for second scenario of IEEE 33-bus for various energy systems.

Parameter	Base case	Case-01	Case-02	Case-03
Cost (million $)	–	40.8	31.25	22.22
APL(KW)	202.7	66.33	77.38	81.05
APLReduction(%)	–	67.3	62	60
Generator/MMR(MW) ,Loc	–	2.09 (24)	1.5 (9)	1 (11)
Generator (MVAR)	–	1.29	0.929	0.619
Solar PV(MW),Loc	–	0.00	0.0651 (33)	0.203 (33)
Wind (MW) ,Loc	–	0.00	0.054 (31)	0.046 (32)
Wind (MVAR)	–	0.00	0.033	0.029
V_MIN_ (p.u.)	0.1931	0.95	0.95	0.95
CO_2_ emission(ton/year)	–	12,821	9198	39.86
CO_2_ Penalty ($)	–	39,772	28,533	123.6

It’s observed of scenario 2 that in case-01, the integration of FFTGs achieves a reduction in NPC to 40.8 million dollars. The relative APL in this case is 66.33KW, and CO_2_ emissions are 12,821 tons/year with a penalty of $39,772. In case-02 for using FFTGs with RESs, NPC decreased to 31.25million dollars. The corresponding APL is 77.38 kW, and the CO_2_ emission is 9198 tons/year with a penalty of $28,533. In case-03, the implementation of N-R HES resulted in a decrease of NPC to 22.22 million dollars. The related APL is 81.05 KW, and CO_2_ emissions are 39.86 tons/year with a penalty of $123.6. It’s observed that the minimum voltages in all cases are the same as shown in Table 7. It’s observed that for the three cases analyzed, Case-01 has the highest Net Present Cost (NPC) value at 40.8 million dollars, while Case-03 has the lowest NPC value, recorded at 22.22 million dollars. Additionally, Case-03 demonstrates the least environmental impact. Specifically, Case-01 emits 12,821 tons of CO2 annually, whereas N-RHES has significantly lower emissions at just 39.86 tons. Consequently, Case-01 incurs the highest penalty for CO2 emissions, while Case-03 faces the lowest penalty. Therefore, when considering both economic (NPC) and environmental (GHG emission) KPIs, Case-03 outperforms the other two energy systems in the IEEE 33-bus radial distribution system.

#### Scenario 3: multi-objective problem (minimizing net present cost, and active power losses)

**Fig. 6:**
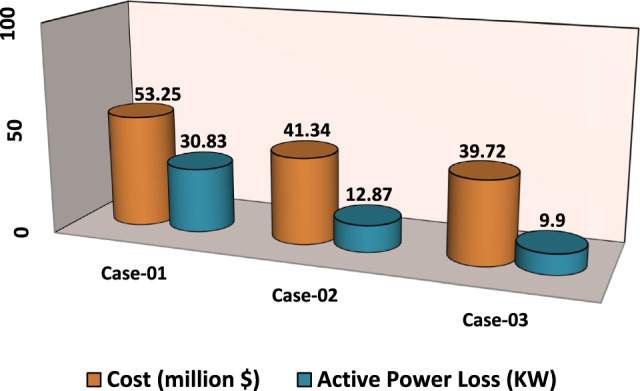
NPC and APL of IEEE 33-bus for third scenario under different energy systems.

In Scenario 3, fuzzy logic and the weighted sum approach are selected to determine the optimal weight of NPC and APL^25^. The effects of unit allocation are evaluated after the installation of distributed generation units, including DG, PV, WTG and MMR. This assessment involves calculating various characteristics like active power losses, net present cost, and minimum bus voltages in the power radial distribution network. Comparison of the effects of all cases is displayed in Table 8. Also in Fig. 6, comparison of the NPC and APL in each case is presented.

**Table 8:** Result of optimization for various energy systems of IEEE 33-bus in terms of APL and NPC.

Parameter	Case-01	Case-02	Case-03
Cost (million $)	53.25	41.34	39.72
APL(KW)	30.83	12.87	9.9
Generator/MMR(MW), Loc	2.6 (6)	1.351 (6)	1 (30)
Generator (MVAR)	1.62	0.837	0.715
Solar PV(MW), Loc	0.00	0.6099 (15)	0.766 (25)
Wind (MW), Loc	0.00	0.7316 (31)	1.012(11)
Wind (MVAR)	0.00	0.4534	0.627
V_MIN_ (p.u)	0.97	0.98	0.992
CO_2_ emission (ton/year)	16,000	8285	45.96
CO_2_ Penalty ($)	49,634	25,700	142.56

To analyze the results of Scenario 3, a simulation was conducted focusing on multi-objective functions. The study examined the impact of different energy system integrations on active power losses (APL), net present cost (NPC), and minimum bus voltages. The findings indicate that in Scenario One, where APL is the primary objective function, APL decreases more significantly than NPC. In Scenario Two, where NPC is the objective function, APL shows a less substantial decrease, while NPC improves to a greater extent. Therefore, it is essential to carry out multi-objective functions to simultaneously reduce both APL and NPC, as demonstrated in Table 8.The comparison of active power losses and net present cost in each case study integrated into the network is given in Fig. 6.

#### Comparison among the proposed hybrid energy systems in terms of APL, NPC and voltage profile.

**Fig. 7:**
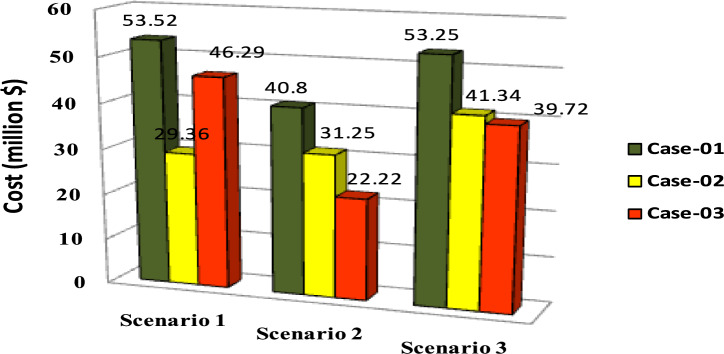
Comparison of NPC of IEEE 33-bus for different energy systems.

**Fig. 8:**
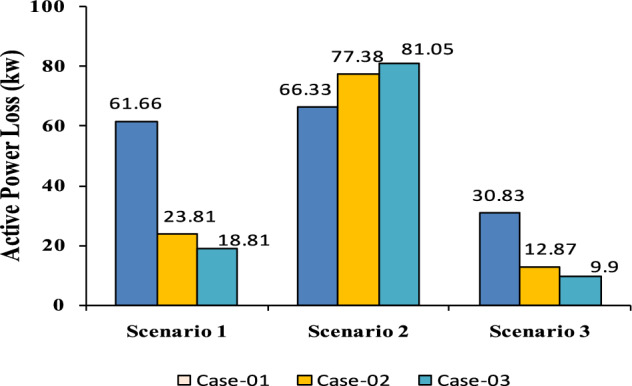
Comparison of APL of IEEE 33-bus for different energy systems.

**Fig. 9:**
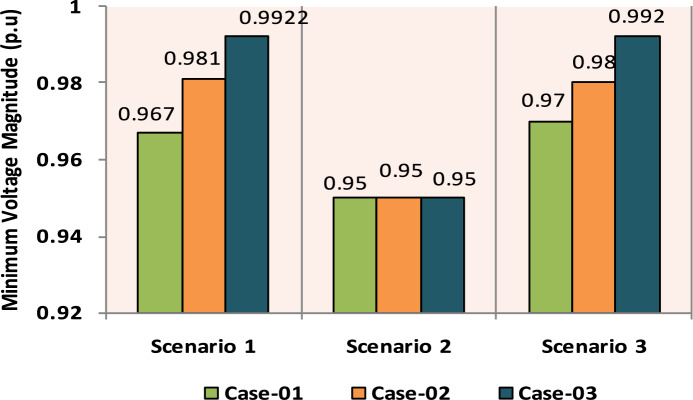
Comparison of Minimum Voltage of IEEE 33-bus for different energy systems.

**Fig. 10:**
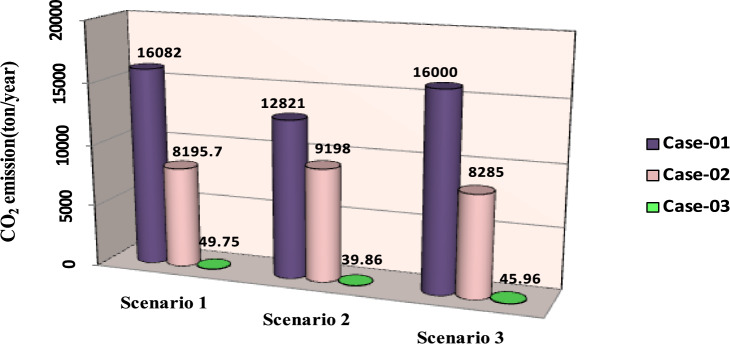
Comparison of CO_2_ emission of IEEE 33-bus for different energy systems.

**Fig. 11:**
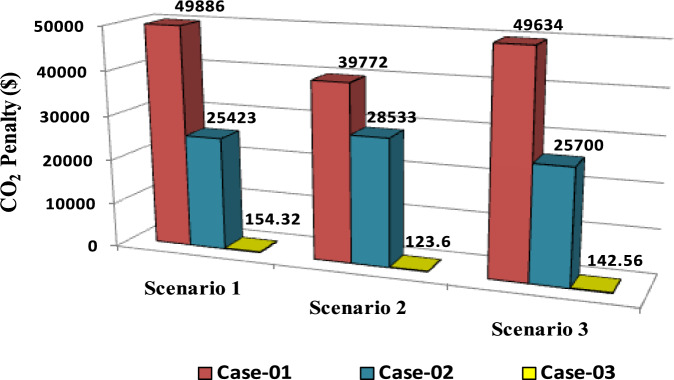
Comparison of CO_2_ Penalty of IEEE 33-bus for different energy systems.

The comparisons of the different scenarios with various energy systems concerning net present cost, active power loss, and bus minimum voltages are shown graphically in Figs. 7, 8, and 9, respectively. The results indicated that Case-03 exhibits the least NPC of all energy systems in scenarios 2 and 3 when concerned with NPC as an objective function. As shown in Fig. 7, NPC is 22.22 million dollars in scenario 2, and 39.7million dollars in scenario 3, while Case-01 has the highest NPC. Also, the results showed that in terms of APL, Case-03 has the lowest APL (18.81 kW) in scenario 1 and (81.05 kW) in scenario 2 and (9.9 kW) in scenario 3, while Case-01 has the highest APL, as in Fig. 8. Additionally, Fig. 9 indicates the significant improvement of the voltage profile, where the minimum voltage magnitudes are 0.967 p.u., 0.95 p.u., and 0.97p.u. in scenarios 1, 2, and 3, respectively. Finally, the proposed N-R HES generates the lowest CO_2_ emissions and incurs a smaller CO_2_ penalty, as shown in Figs. 10 and 11, respectively. From the findings, it is evident that in scenario 3, Case-03 emerges as the most effective energy system for the IEEE 33-Bus, demonstrating excellence in technical, economic, and environmental KPIs.

## IEEE 69-bus radial distribution system

### Scenario 1: active power loss minimization (single-objective problem)

**Fig. 12:**
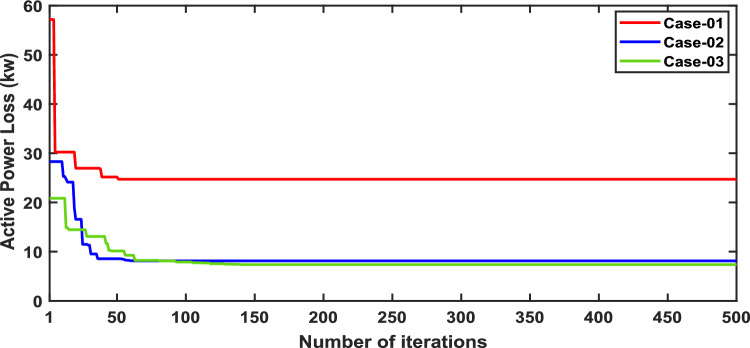
Convergence characteristics of IEEE 69-Bus for scenario 1 for Case-01, Case-02 and Case-03.

**Fig. 13:**
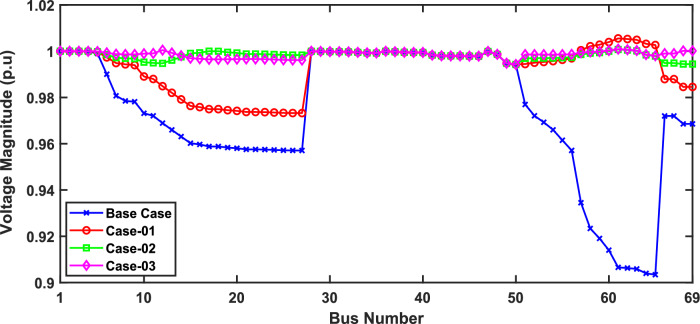
Voltage profile for first scenario of IEEE 69-bus under different case studies.

The optimal placement of (DG, PV, WTG, and MMR) units was used in scenario 1 in order to reduce the total active power loss of the three energy systems. Table 9 displays the effects of every case using the proposed approach. Additionally, Fig. 12 shows the convergence characteristic of the suggested approach. The voltage profile of different energy systems integration is displayed in Fig. 13.

**Table 9:** Result of optimization for various energy systems of IEEE 69-bus in terms of APL.

Parameter	Base Case	Case-01	Case-02	Case-03
APL (KW)	238.8	24.70	8.13	7.35
APL Reduction (%)	–	90	96	97
Cost (million $)	–	40.81	44.26	52.77
Generator/MMR(MW), Loc	–	2 (61)	1.806 (61)	1.76 (61)
Generator (MVAR)		1.239	1.119	1.094
Solar PV(MW), Loc	–	0.00	0.00	0.246(21)
Wind (MW), Loc	–	0.00	0.536 (17)	0.631 (12)
Wind (MVAR)	–	0.00	0.332	0.391
V_MIN_ (p.u)	0.9034	0.973	0.994	0.994
CO_2_ emission (ton/year)	–	12,264	11,074	70.34
CO_2_ Penalty ($)	–	38,043	34,351	218.19

Form the analysis for scenario 1 results, according to Table 9 in the base case, the minimum voltage is 0.9034 p.u, and the active power loss is 238.8 kW. In case-01, the implementation of FFTGs achieves improvement in APL that decreases to 24.7 KW with a reduction of 90%, and the minimum voltage is 0.973. The cost in this case is 40.8 million dollars, and CO_2_ emissions are 12,264 tons/year with a penalty of 38,043 dollars. After introducing FFTGs with RESs in Case-02, the APL reduces to 8.13 KW with a reduction of 96%, and the minimum voltage is 0.994. The relative cost in this case is 44.3 million dollars, and CO_2_ emissions are 11,074 tons/year with a penalty of 34,351 dollars. In the third case, for the integration of N-R HES, APL reduces to 7.35 KW with reductions of 97% and the minimum voltage to 0.994. The corresponding cost is 52.77 million $ and CO_2_ emission is 70.34 tons/year with a penalty of 218.9 $. It’s observed that for the three cases, Case-03 demonstrates the highest APLRat 97%, while Case-01 shows the lowest APLR at 90%. Annually, Case-03 emits only 70.34 tons of CO2 from the N-RHES, compared to 12,264 tons from Case-01. This indicates that Case-03 has the least environmental impact based on these factors. Consequently, Case-01 incurs the largest penalty for CO2 emissions, whereas Case-03 faces the lowest penalty. Therefore, with respect to technical (APL) and environmental (GHG emissions) KPIs, Case-03 surpasses the other two energy systems in the IEEE 69-bus radial distribution system.

#### Scenario 2: optimizing single-objective problem (net present cost)

In this scenario, the net present cost is minimized for IEEE 69-Bus using the WSO algorithm. The comparisons of the results of integration of DG, PV, WTG, and MMR in each of the aforementioned.

**Fig. 14:**
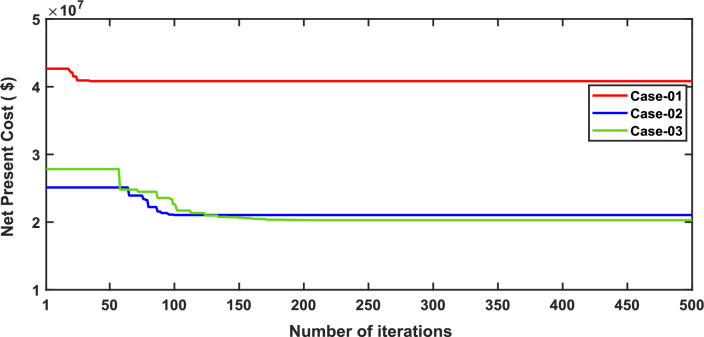
Convergence characteristics of IEEE 69-bus for scenario 1 for Case-01, Case-02 and Case-03.

**Fig. 15:**
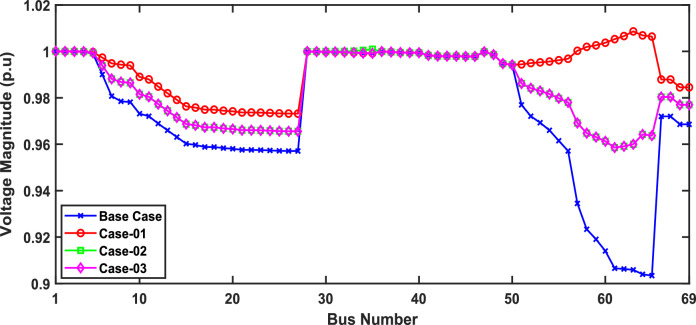
Voltage profile of IEEE 69-bus for second scenario under various case studies.

Cases are shown in Table 10. Figures 14 and 15 present the convergence graph and voltage profile comparison for the different energy systems, respectively.

**Table 10:** Optimization result for various energy systems of IEEE 69-bus in terms of NPC.

Parameter	Case-01	Case-02	Case-03
Cost (million $)	40.8	21.05	20.28
APL(KW)	24.72	63.51	66.79
Generator/MMR(MW), Loc	2.09 (49)	1.007 (65)	1 (62)
Generator (MVAR)	1.296	0.624	0.6197
Solar PV(MW), Loc	0.00	0.00	0.0013 (66)
Wind (MW), Loc	0.00	0.114 (26)	0.0034 (29)
Wind (MVAR)	0.00	0.0707	0.0021
V_MIN_ (p.u)	0.973	0.9557	0.9586
CO_2_ emission (ton/year)	12,821	6174	39.86
CO_2_ Penalty ($)	39,772	19,153	123.64

For results analysis of scenario 2, it’s observed that in the first case, the introduction of FFTGs resulted in decreasing of NPC to 40.8 milliondollars. The corresponding APL in this case is 24.72 KW and CO_2_ emission is 12,821 tons/year with a penalty of $39,772. In the second case for the implementation of FFTGs with RESs, NPC decreased to 21.05 milliondollars. The related APL is 63.51 KW and CO_2_ emission is 6174 tons/year with a penalty $19,153. In the third case, the integration of N-R HES achieves a reduction in NPC to 20.28million dollars. The relative APL is 66.79 KW and CO_2_ emission is 39.86 tons/year with a penalty of $123.64. It’s observed that the minimum voltages in all cases are 0.973 p.u., 0.956 (p.u.), and 0.959(p.u.), respectively. The convergence curve and the voltage magnitude for all the cases are displayed in Figs. 14 and 15, respectively. It’s observed that from the three cases analyzed, Case-01 has the largest NPC value at 40.8 million dollars, while Case-03 has the lowest NPC value at 20.28 million dollars. Regarding environmental impact, Case-03 produces the least CO_2_, releasing only 39.86 tons annually from the N-RHES system. In contrast, Case-01 emits a significantly greater amount of CO_2_. Consequently, the penalties for CO_2_ emissions are lowest for Case-03 and highest for Case-01. Therefore, Case-03 excels over the other two energy systems in the IEEE 69-bus radial distribution system when evaluating environmental (GHG) and economic (NPC) KPIs.

### Scenario3: multi-objective problem (minimizing net present cost and active power losses,)

**Fig. 16:**
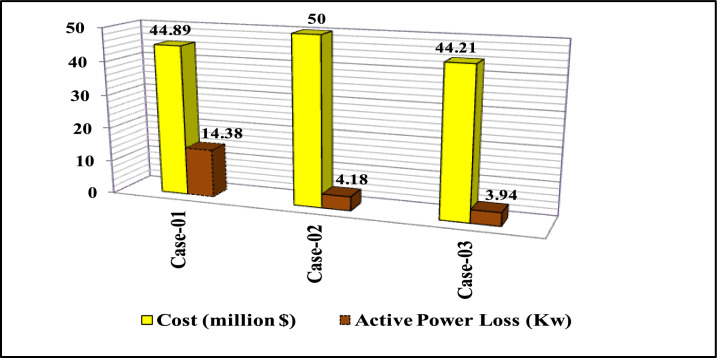
NPC and APL of IEEE 69-Bus for third scenario under various case studies.

This scenario employs WSO for optimizing the placement of (DG, PV, WTG, and MMR) units under multi-objective functions and constraints. Table 11 displays the results of different energy systems integration. Also, the comparison of the APL and NPC of all cases is given in Fig. 16.

**Table 11:** Optimization result for various energy systems of IEEE 69-bus in terms of APL and NPC.

Parameter	Case-01	Case-02	Case-03
Cost (million $)	44.89	50	44.21
APL(KW)	14.38	4.18	3.94
Generator/MMR(MW), Loc	2.2 (61)	1.868 (61)	1.83 (61)
Generator (MVAR)	1. 36	1.158	1.135
Solar PV(MW), Loc	0.00	0.780 (4)	0.780 (48)
Wind (MW), Loc	0.00	0.564 (17)	0. 576 (17)
Wind (MVAR)	0.00	0.349	0.357
V_MIN_ (p.u)	0.975	0.994	0.995
CO_2_ emission (ton/year)	13,490	11,454	73.02
CO_2_ Penalty ($)	41,848	35,532	226.50

For results analysis of scenario 3, it’s observed that in Case-03, the integration of N-R HES achieves improvement in both NPC 44.21 million dollars) and APL (3.94kW) compared with Case-02, the NPC (50 million dollars) and APL (4.18 kW) and Case 01, which NPC (44.89 million dollars) and APL (14.38 kW).

#### Comparison among the integrated energy systems in terms of APL, NPC and voltage profile.

**Fig. 17:**
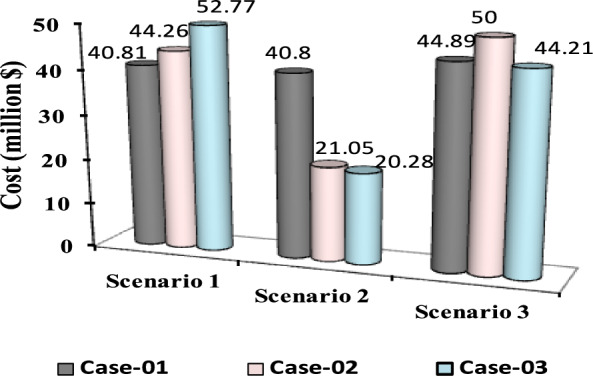
Comparison of NPC of IEEE 69-bus for different energy systems.

**Fig. 18:**
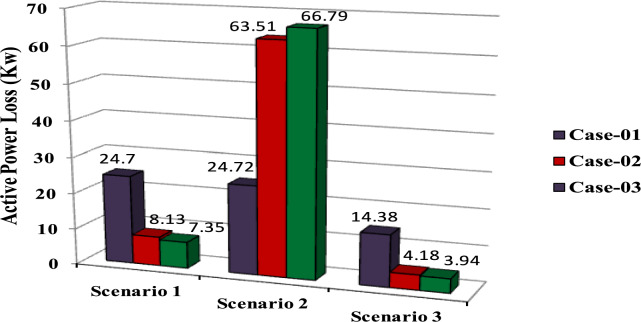
Comparison of APL of IEEE 69-bus for different energy systems.

**Fig. 19:**
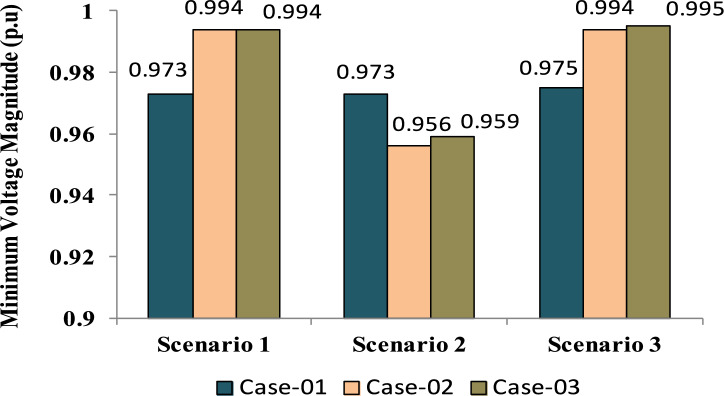
Comparison of Minimum Voltage of IEEE 69-bus for different energy systems.

**Fig. 20:**
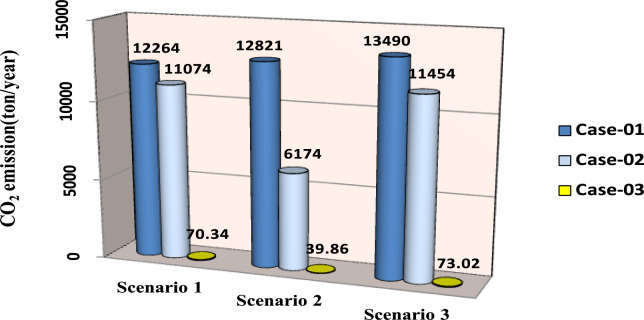
Comparison of CO_2_ emission of IEEE 69-bus for different energy systems.

**Fig. 21:**
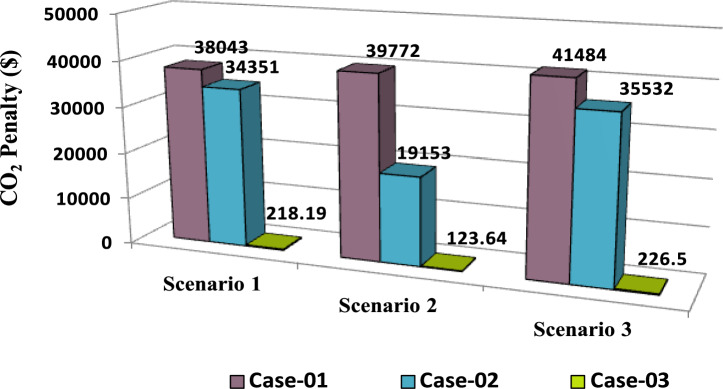
Comparison of CO_2_ Penalty of IEEE 69-bus for different energy systems.

The comparisons of the different scenarios with various energy systems in terms of net present cost, bus minimum voltages, and active power loss are displayed graphically in Figs. 17, 19, and 18, respectively. In the third scenario, N-R HES exhibits the lowest NPC and APL within the three energy systems. As shown in Fig. 16, NPC is 44.21 million dollarsand APL (3.94 kW). Additionally, Fig. 19 indicates the significant improvement of the voltage profile, where the minimum voltage magnitude is 0.995 (p.u.).Ultimately, the proposed N-R HES produces the least CO_2_ emissions and incurs a lower CO_2_ penalty, as illustrated in Figs. 20 and 21, respectively. Results indicate that Case-03, which excels in technical, financial, and environmental KPIs, is the most efficient energy system for the IEEE 69-Bus in scenario three.

## Conclusion

Large-scale nuclear power plants are not a new concept. However, due to their high capital costs and associated risks, the implementation of small-scale nuclear power plants has been proposed as a more viable alternative. In this study, the White Shark Optimizer (WSO) is employed to determine the optimal sizing and placement of various Hybrid Energy Systems (HES), including a Stand-alone Fossil Fuel Energy System, a Fossil Fuel–Renewable Hybrid Energy System, and a Nuclear–Renewable Hybrid Energy System. These configurations are optimized to achieve both single- and multi-objective functions aimed at improving voltage profiles, reducing power losses, and minimizing costs and greenhouse gas (GHG) emissions.

To validate the proposed approach, a comprehensive performance analysis is conducted using the IEEE 33-bus and IEEE 69-bus radial distribution systems. Three key performance indicators Net Present Cost (NPC), Active Power Loss (APL), and GHG emissions are used to compare the different scenarios. The results demonstrate that the combination of nuclear and renewable energy sources offers the most effective solution for sustainable energy generation and decarbonization. Compared to other configurations, the Nuclear–Renewable HES (N-R HES) achieves the lowest NPC and APL while emitting the least amount of CO₂.For the IEEE 33-bus system, the best scenario involving multi-objective optimization reduces power losses to 9.9 (kW) and NPC to 39.72 (million $), while improving the minimum voltage magnitude from 0.9131 to 0.992 p.u. The system also generates the lowest CO₂ emissions (45.96 tons/year) and incurs a reduced CO₂ penalty of $142.56.

Similarly, for the IEEE 69-bus system, the N-R HES achieves the lowest APL (3.94 kW) and NPC (44.21 million $), along with a significant improvement in voltage profile, where the minimum voltage magnitude reaches 0.995 p.u. Ultimately, the proposed N-R HES produces the lowest CO₂ emissions (73.02 tons/year) and incurs a smaller CO₂ penalty of $226.50.

These findings prove that integrating nuclear and renewable energy sources in a hybrid system is both technically and economically feasible and may represent the most effective strategy for eliminating emissions in radial distribution networks.

The main findings of this study are summarized as follows:

- The study explores the feasibility and advantages of incorporating micro nuclear reactors, into hybrid energy systems for reliable and sustainable electricity distribution.
- Mathematical models for various HES configurations are developed to enhance the technical, economic, and environmental performance of distribution networks by solving both single and multi-objective optimization problems.
- A novel metaheuristic algorithm, the White Shark Optimizer, is applied for the first time to find the optimal configuration of HES for improving minimizing power losses, voltage profiles, reducing costs, and lowering greenhouse gas emissions.
- Results prove that the N-R HES provides the most effective among cost, environmental impact, and system performance.

## Future work

- Despite the promising results, the current evaluation is limited to standard test distribution networks. Future research should extend the analysis to real-world, large-scale distribution systems to better assess the practical feasibility and performance of Nuclear-Renewable Hybrid Energy Systems (N-R HES).
- The models assume steady-state operation. Therefore, future work could expand the optimization framework by considering transient events or system faults, which may significantly impact system performance.
- Finally, the study does not account for the influence of energy storage systems. Integrating emerging technologiessuch as advanced energy storage systems into future studies could further improve operational sustainability, flexibility, and efficiency.

## Supplementary Information

Supplementary Information.

## Data Availability

The datasets generated and/or analyzed during the current study are available from the corresponding author upon reasonable request.

## Abbreviations

APL: Active power losses
APLR: Active power losses reduction
CHP: Combined heat and power
FFTGs: Fossil fuel-based thermal generators
GHG: Greenhouse gas generation
HES: Hybrid energy systems
IAEA: International atomic energy agency
IMF: International monetary fund
KPIs: Key performance indicators
MMR: Micro modular reactor
MOF: Multiple objective function
NPPs: Nuclear power plants
NPC: Net present cost
N-R HES: Nuclear-renewable hybrid energy system
O&M: Operating and maintenance
PV: Photo voltaic
RESs: Renewable energy sources
SMRs: Small modular reactors
WSO: White shark optimizer
WTG: Wind turbine generator
