# Supplementary material for: Optimal planning of integrated nuclear-hybrid renewable energy systems for electrical distribution networks based on artificial intelligence
Source: Sci Rep. 2025 Jul 17;15:26004. doi: 10.1038/s41598-025-11049-z (PMC12271530; doi:10.1038/s41598-025-11049-z)
Supplement: Supplementary file 1 — Supplementary Information. [file 41598_2025_11049_MOESM1_ESM.docx]

**Supplementary File: Study Methodology and Simulation Workflow**

Optimal Planning of Integrated Nuclear- Hybrid Renewable Energy Systems for Electrical Distribution Networks Based on Artificial Intelligence

Samira M. Nassar^1🖂^ ,A.A.Saleh ^1^ ,Ayman A. Eisa^1^, E.M.Abdallah^2^, Ibrahim A. Nassar^2^

^1^ Department of Nuclear Safety and Radiological Emergencies, NCRRT ,Egyptian Atomic Energy Authority, Egypt. ^2^ Department of Electrical Engineering, Faculty of Engineering, Al-AzharUniversity, Cairo, Egypt.^🖂^Samira.nassar@eaea.org.eg

**Overview of Study Steps**

This research focuses on the modeling and optimization of various Hybrid Energy Systems (HES) using both single- and multi-objective optimization frameworks. The single-objective function targets the minimization of Net Present Cost (NPC), while also improving voltage profiles and reducing Active Power Loss (APL). In contrast, the multi-objective optimization concurrently addresses both economic (NPC) and technical (APL and voltage stability) parameters. This integrated approach marks a novel contribution by bridging economic and technical criteria, setting it apart from existing studies which typically focus only on cost minimization.

A comprehensive comparison of three HES configurations is carried out using multiple techno-economic key performance indicators (KPIs), such as APL, greenhouse gas (GHG) emissions, and NPC. Simulations are conducted on two standard radial distribution systems—IEEE 33-bus and IEEE 69-bus networks. The results show that the Nuclear–Renewable HES (N-R HES) achieves superior performance by significantly reducing GHG emissions and improving both economic and technical metrics.

For clarity, the study follows the stepwise process illustrated in **Figure S1**.

Step1: Mathematical model of three hybrid energy systems.

Step2:Propose a meta_heuristic algorithm to obtain the optimal location and size of the three hybrid energy resources.

Step3: Minimize Active Power Loss (APL) as an objective function.

Step 4 and minimize Net Present Cost (NPC) as second objective function.

Step 5: Apply multi -objective function to minimize APL, NPC and improve voltage profile.

**Figure S1: Workflow of the Study**

**Step 1: Development of Mathematical Models for HES Configurations**

Three different HES models are formulated and applied to the IEEE 33-bus and IEEE 69-bus test systems. These systems are analyzed in their base configurations (prior to energy resource integration) to enable comparative assessment.

- **IEEE 33-bus system**: 3.72 MW, 2.3 MVAR
- **IEEE 69-bus system**: 3.89 MW, 2.6936 MVAR, 12.6 kV

**Step 2: Introduction of a Metaheuristic Optimization Algorithm**

The White Shark Optimizer (WSO), a metaheuristic algorithm, is employed to determine the optimal locations and sizes of distributed energy resources. The algorithm solves both single- and multi-objective problems aimed at minimizing power losses and total NPC.

**Step 3: Objective Function 1 – Minimization of Active Power Loss (APL)**

Minimizing power losses is essential for enhancing the efficiency of distributed energy systems such as photovoltaic (PV) and wind turbine generators (WTG). The total APL is calculated using the following equation:

$$P_{\mathrm{Tloss}}=\sum_{K_{(\mathrm{mn})}=1}^{n_{\mathrm{br}}} R_{(K)}\left[ \frac{P_{(n)}^{2}+Q_{(n)}^{2}}{\left| V_{(n)} \right|^{2}} \right] (S1)$$

Where:

- $P_{\mathrm{Tloss}}$is the total active power loss;
- k is the index of a branch connected between buses n and m;
- P_(n)_ , Q_(n)_ are the real and reactive power loads ;
- V_(n)_ is the voltage magnitude of n^th^ bus; n_br_ is the number of network branches.

**Step 4: Objective Function 2 – Minimization of Net Present Cost (NPC)**

The NPC represents the economic evaluation metric and serves as the second objective function. It is calculated as:

$\min f_{\mathrm{NPC}}=\sum_{j\epsilon k} \mathrm{NPC}_{j}$ (S2)

$$\mathrm{NPC}_{j}=C_{cap,j}+C_{O\&M,j}+{C_{fuelc,j}+C}_{rep,j}-C_{salv,j} \left( S3 \right)$$

Where:

- $C_{cap,j}$: Capital cost;
- $C_{O\&M,j}$: Operation and maintenance cost;
- $C_{fuelc,j}$: Fuel cost;
- $C_{rep,j}:$ Replacement cost;
- $C_{salv,j}:$ Salvage value.

**Step 5: Multi-Objective Optimization – Combined APL and NPC Minimization**

To balance the dual objectives, the **Weighted Sum Method** is adopted:

$$OF=min\left( F \right)=w_{1}\frac{P_{L}^{with DG}}{P_{L}^{no DG}}+w_{2}\frac{\mathrm{NPC}^{with DG}}{\mathrm{NPC}^{no DG}} , w_{1}+w_{2}=1 (S4)$$

where,

- $w_{1}$ and $w_{2}$ are total power loss and NPC weighting coefficients, respectively;
- $P_{L}^{with DG}\mathrm{and}\mathrm{NPC}^{with DG}$ refer to net present cost and active power loss after energy resources installation;
- $\mathrm{NPC}^{no DG}\mathrm{and}P_{L}^{no DG}$ refer to net present cost and active power loss before energy resource integration.

**Step 6: Simulation on IEEE Radial Distribution Networks**

The complete optimization and simulation process is executed in MATLAB. The three HES models are applied to the IEEE 33-bus and IEEE 69-bus systems.

- **Bus and line data for IEEE 33-bus system**: See **Appendix A**
- **Bus and line data for IEEE 69-bus system**: See **Appendix B**

**Appendices**

**Appendix A** – Bus and Line Data for IEEE 33-Bus Radial System
**Appendix B** – Bus and Line Data for IEEE 69-Bus Radial System

(*Detailed tables provided in the original supplementary material*)

**Appendix A: IEEE 33-Bus Test Radial System Data [1]**

**Table A.1: Bus Data of 33-Bus System Table A.2: Line Data of 33-Bus System**

| **Bus No.** | **Bus Type** | **V(0)** | **delta(0)** | **Load (MW)** | **Load (Mvar)** |  | From Bus | To Bus | R (p.u.) | X (p.u.) |
| --- | --- | --- | --- | --- | --- | --- | --- | --- | --- | --- |
| 1 | 1 | 1 | 0 | 0 | 0 |  | 1 | 2 | 0.0006 | 0.0003 |
| 2 | 0 | 1 | 0 | 0.1 | 0.06 |  | 2 | 3 | 0.0031 | 0.0016 |
| 3 | 0 | 1 | 0 | 0.09 | 0.04 |  | 3 | 4 | 0.0023 | 0.0012 |
| 4 | 0 | 1 | 0 | 0.12 | 0.08 |  | 4 | 5 | 0.0024 | 0.0012 |
| 5 | 0 | 1 | 0 | 0.06 | 0.03 |  | 5 | 6 | 0.0051 | 0.0044 |
| 6 | 0 | 1 | 0 | 0.06 | 0.02 |  | 6 | 7 | 0.0012 | 0.0039 |
| 7 | 0 | 1 | 0 | 0.2 | 0.1 |  | 7 | 8 | 0.0107 | 0.0077 |
| 8 | 0 | 1 | 0 | 0.2 | 0.1 |  | 8 | 9 | 0.0064 | 0.0046 |
| 9 | 0 | 1 | 0 | 0.06 | 0.02 |  | 9 | 10 | 0.0063 | 0.0046 |
| 10 | 0 | 1 | 0 | 0.06 | 0.02 |  | 10 | 11 | 0.0012 | 0.0004 |
| 11 | 0 | 1 | 0 | 0.045 | 0.03 |  | 11 | 12 | 0.0023 | 0.0008 |
| 12 | 0 | 1 | 0 | 0.06 | 0.035 |  | 12 | 13 | 0.0092 | 0.0072 |
| 13 | 0 | 1 | 0 | 0.06 | 0.035 |  | 13 | 14 | 0.0034 | 0.0044 |
| 14 | 0 | 1 | 0 | 0.12 | 0.08 |  | 14 | 15 | 0.0037 | 0.0033 |
| 15 | 0 | 1 | 0 | 0.06 | 0.01 |  | 15 | 16 | 0.0047 | 0.0034 |
| 16 | 0 | 1 | 0 | 0.06 | 0.02 |  | 16 | 17 | 0.008 | 0.0107 |
| 17 | 0 | 1 | 0 | 0.06 | 0.02 |  | 17 | 18 | 0.0046 | 0.0036 |
| 18 | 0 | 1 | 0 | 0.09 | 0.04 |  | 2 | 19 | 0.001 | 0.001 |
| 19 | 0 | 1 | 0 | 0.09 | 0.04 |  | 19 | 20 | 0.0094 | 0.0085 |
| 20 | 0 | 1 | 0 | 0.09 | 0.04 |  | 20 | 21 | 0.0026 | 0.003 |
| 21 | 0 | 1 | 0 | 0.09 | 0.04 |  | 21 | 22 | 0.0044 | 0.0058 |
| 22 | 0 | 1 | 0 | 0.09 | 0.04 |  | 3 | 23 | 0.0028 | 0.0019 |
| 23 | 0 | 1 | 0 | 0.42 | 0.05 |  | 23 | 24 | 0.0056 | 0.0044 |
| 24 | 0 | 1 | 0 | 0.42 | 0.2 |  | 24 | 25 | 0.0056 | 0.0044 |
| 25 | 0 | 1 | 0 | 0.06 | 0.2 |  | 6 | 26 | 0.0013 | 0.0006 |
| 26 | 0 | 1 | 0 | 0.06 | 0.025 |  | 26 | 27 | 0.0018 | 0.0009 |
| 27 | 0 | 1 | 0 | 0.06 | 0.025 |  | 27 | 28 | 0.0066 | 0.0058 |
| 28 | 0 | 1 | 0 | 0.12 | 0.02 |  | 28 | 29 | 0.005 | 0.0044 |
| 29 | 0 | 1 | 0 | 0.2 | 0.07 |  | 29 | 30 | 0.0032 | 0.0016 |
| 30 | 0 | 1 | 0 | 0.15 | 0.6 |  | 30 | 31 | 0.0061 | 0.006 |
| 31 | 0 | 1 | 0 | 0.21 | 0.07 |  | 31 | 32 | 0.0019 | 0.0023 |
| 32 | 0 | 1 | 0 | 0.06 | 0.1 |  | 32 | 33 | 0.0021 | 0.0033 |
| 33 | 0 | 1 | 0 | 0.06 | 0.04 |  |  |  |  |  |

**Appendix B: IEEE 69-Bus Test Radial System Data [2]**

**Table B.1: Bus Data of 69-Bus System Table B.2: Line Data of 69-Bus System**

| **Bus No.** | **Bus Type** | **V(0)** | **delta(0)** | **Load (MW)** | **Load (Mvar )** |  | **From Bus** | **To Bus** | **R (p.u.)** | **X (p.u.)** |
| --- | --- | --- | --- | --- | --- | --- | --- | --- | --- | --- |
| 1 | 1 | 1 | 0 | 0 | 0 |  | 1 | 2 | 3.10E-06 | 7.50E-06 |
| 2 | 0 | 1 | 0 | 0 | 0 |  | 2 | 3 | 3.10E-06 | 7.50E-06 |
| 3 | 0 | 1 | 0 | 0 | 0 |  | 3 | 4 | 9.40E-06 | 2.25E-05 |
| 4 | 0 | 1 | 0 | 0 | 0 |  | 4 | 5 | 0.000134 | 0.000183 |
| 5 | 0 | 1 | 0 | 0 | 0 |  | 5 | 6 | 0.002284 | 0.001163 |
| 6 | 0 | 1 | 0 | 0.0026 | 0.0022 |  | 6 | 7 | 0.002377 | 0.001211 |
| 7 | 0 | 1 | 0 | 0.0403 | 0.03 |  | 7 | 8 | 0.000575 | 0.000293 |
| 8 | 0 | 1 | 0 | 0.075 | 0.054 |  | 8 | 9 | 0.000308 | 0.000157 |
| 9 | 0 | 1 | 0 | 0.03 | 0.022 |  | 9 | 10 | 0.00511 | 0.001689 |
| 10 | 0 | 1 | 0 | 0.028 | 0.019 |  | 10 | 11 | 0.001168 | 0.000386 |
| 11 | 0 | 1 | 0 | 0.145 | 0.104 |  | 11 | 12 | 0.004439 | 0.001467 |
| 12 | 0 | 1 | 0 | 0.145 | 0.104 |  | 12 | 13 | 0.006426 | 0.002121 |
| 13 | 0 | 1 | 0 | 0.008 | 0.005 |  | 13 | 14 | 0.006514 | 0.002121 |
| 14 | 0 | 1 | 0 | 0.008 | 0.005 |  | 14 | 15 | 0.006601 | 0.002181 |
| 15 | 0 | 1 | 0 | 0 | 0 |  | 15 | 16 | 0.001227 | 0.000406 |
| 16 | 0 | 1 | 0 | 0.045 | 0.03 |  | 16 | 17 | 0.002336 | 0.000772 |
| 17 | 0 | 1 | 0 | 0.06 | 0.035 |  | 17 | 18 | 2.93E-05 | 1.00E-05 |
| 18 | 0 | 1 | 0 | 0.06 | 0.035 |  | 18 | 19 | 0.002044 | 0.000676 |
| 19 | 0 | 1 | 0 | 0 | 0 |  | 19 | 20 | 0.001314 | 0.000431 |
| 20 | 0 | 1 | 0 | 0.001 | 0.0006 |  | 20 | 21 | 0.002131 | 0.000704 |
| 21 | 0 | 1 | 0 | 0.114 | 0.081 |  | 21 | 22 | 8.73E-05 | 2.87E-05 |
| 22 | 0 | 1 | 0 | 0.005 | 0.0035 |  | 22 | 23 | 0.000993 | 0.000328 |
| 23 | 0 | 1 | 0 | 0 | 0 |  | 23 | 24 | 0.002161 | 0.000714 |
| 24 | 0 | 1 | 0 | 0.028 | 0.02 |  | 24 | 25 | 0.004672 | 0.001544 |
| 25 | 0 | 1 | 0 | 0 | 0 |  | 25 | 26 | 0.001927 | 0.000637 |
| 26 | 0 | 1 | 0 | 0.014 | 0.01 |  | 26 | 27 | 0.001081 | 0.000357 |
| 27 | 0 | 1 | 0 | 0.014 | 0.01 |  | 3 | 28 | 2.75E-05 | 6.74E-05 |
| 28 | 0 | 1 | 0 | 0.026 | 0.0186 |  | 28 | 29 | 0.000399 | 0.000976 |
| 29 | 0 | 1 | 0 | 0.026 | 0.0186 |  | 29 | 30 | 0.002482 | 0.000821 |
| 30 | 0 | 1 | 0 | 0 | 0 |  | 30 | 31 | 0.000438 | 0.000145 |
| 31 | 0 | 1 | 0 | 0 | 0 |  | 31 | 32 | 0.00219 | 0.000724 |
| 32 | 0 | 1 | 0 | 0 | 0 |  | 32 | 33 | 0.005235 | 0.001757 |
| 33 | 0 | 1 | 0 | 0.01 | 0.01 |  | 33 | 34 | 0.010657 | 0.003523 |
| 34 | 0 | 1 | 0 | 0.014 | 0.014 |  | 34 | 35 | 0.009197 | 0.00304 |
| 35 | 0 | 1 | 0 | 0.004 | 0.004 |  | 3 | 36 | 2.75E-05 | 6.74E-05 |
| 36 | 0 | 1 | 0 | 0.026 | 0.01855 |  | 36 | 37 | 0.000399 | 0.000976 |
| 37 | 0 | 1 | 0 | 0.026 | 0.01855 |  | 37 | 38 | 0.000657 | 0.000767 |
| 38 | 0 | 1 | 0 | 0 | 0 |  | 38 | 39 | 0.00019 | 0.000222 |
| 39 | 0 | 1 | 0 | 0.024 | 0.017 |  | 39 | 40 | 1.12E-05 | 1.31E-05 |
| 40 | 0 | 1 | 0 | 0.024 | 0.017 |  | 40 | 41 | 0.004544 | 0.005309 |
| 41 | 0 | 1 | 0 | 0.102 | 0.001 |  | 41 | 42 | 0.001934 | 0.002261 |
| 42 | 0 | 1 | 0 | 0 | 0 |  | 42 | 43 | 0.000256 | 0.000298 |
| 43 | 0 | 1 | 0 | 0.006 | 0.0043 |  | 43 | 44 | 5.74E-05 | 7.24E-05 |
| 44 | 0 | 1 | 0 | 0 | 0 |  | 44 | 45 | 0.00068 | 0.000857 |
| 45 | 0 | 1 | 0 | 0.03922 | 0.0263 |  | 45 | 46 | 5.60E-06 | 7.50E-06 |
| 46 | 0 | 1 | 0 | 0.03922 | 0.0263 |  | 4 | 47 | 2.12E-05 | 5.24E-05 |
| 47 | 0 | 1 | 0 | 0 | 0 |  | 47 | 48 | 0.000531 | 0.0013 |
| 48 | 0 | 1 | 0 | 0.079 | 0.0564 |  | 48 | 49 | 0.001808 | 0.004424 |
| 49 | 0 | 1 | 0 | 0.3847 | 0.2745 |  | 49 | 50 | 0.000513 | 0.001255 |
| 50 | 0 | 1 | 0 | 0.3847 | 0.2745 |  | 8 | 51 | 0.000579 | 0.000295 |
| 51 | 0 | 1 | 0 | 0.0405 | 0.0283 |  | 51 | 52 | 0.002071 | 0.000711 |
| 52 | 0 | 1 | 0 | 0.0036 | 0.0027 |  | 9 | 53 | 0.001086 | 0.000553 |
| 53 | 0 | 1 | 0 | 0.00435 | 0.0035 |  | 53 | 54 | 0.001267 | 0.000645 |
| 54 | 0 | 1 | 0 | 0.0264 | 0.019 |  | 54 | 55 | 0.001755 | 0.000903 |
| 55 | 0 | 1 | 0 | 0.024 | 0.0172 |  | 55 | 56 | 0.001755 | 0.000894 |
| 56 | 0 | 1 | 0 | 0 | 0 |  | 56 | 57 | 0.00992 | 0.00333 |
| 57 | 0 | 1 | 0 | 0 | 0 |  | 57 | 58 | 0.00489 | 0.001641 |
| 58 | 0 | 1 | 0 | 0 | 0 |  | 58 | 59 | 0.001898 | 0.000628 |
| 59 | 0 | 1 | 0 | 0.1 | 0.072 |  | 59 | 60 | 0.002409 | 0.000731 |
| 60 | 0 | 1 | 0 | 0 | 0 |  | 60 | 61 | 0.003166 | 0.001613 |
| 61 | 0 | 1 | 0 | 1.244 | 0.888 |  | 61 | 62 | 0.000608 | 0.00031 |
| 62 | 0 | 1 | 0 | 0.032 | 0.023 |  | 62 | 63 | 0.000905 | 0.000461 |
| 63 | 0 | 1 | 0 | 0 | 0 |  | 63 | 64 | 0.004433 | 0.002258 |
| 64 | 0 | 1 | 0 | 0.227 | 0.162 |  | 64 | 65 | 0.006495 | 0.003308 |
| 65 | 0 | 1 | 0 | 0.059 | 0.042 |  | 11 | 66 | 0.001255 | 0.000381 |
| 66 | 0 | 1 | 0 | 0.018 | 0.013 |  | 66 | 67 | 2.93E-05 | 8.70E-06 |
| 67 | 0 | 1 | 0 | 0.018 | 0.013 |  | 12 | 68 | 0.004613 | 0.001525 |
| 68 | 0 | 1 | 0 | 0.028 | 0.02 |  | 68 | 69 | 2.93E-05 | 1.00E-05 |
| 69 | 0 | 1 | 0 | 0.028 | 0.02 |  |  |  |  |  |

**Note**

This supplementary document provides detailed insights and methodological explanations complementing the main paper:

**“Optimal Planning of Integrated Nuclear–Hybrid Renewable Energy Systems for Electrical Distribution Networks Based on Artificial Intelligence”**

**References**

1. E. M. Abdallah, M. I. Elsayed, M. M. ELgazzer, Amal A. H., "Coyote multi-objective optimization algorithm for optimal location and sizing of renewable distributed generators," *Int. J. Electr. Comput. Eng.*, vol. 11, no. 2, pp. 975–983, 2021. DOI: 10.11591/ijece.v11i2.pp975-983
2. E. M. Abdallah, M. I. Elsayed, M. M. ELgazzer, Amal A. H., "Optimal Location and Sizing of Renewable Distributed Generators in Radial Distribution System Using Coyote Optimization Algorithm," *Int. J. Adv. Sci. Technol.*, pp. 10633–10643, 2020.
